# *In vitro* biological action of aqueous extract from roots of *Physalis angulata* against *Leishmania (Leishmania) amazonensis*

**DOI:** 10.1186/s12906-015-0717-1

**Published:** 2015-07-24

**Authors:** Raquel Raick P. da Silva, Bruno J. M. da Silva, Ana Paula D. Rodrigues, Luis Henrique S. Farias, Milton N. da Silva, Danila Teresa V. Alves, Gilmara N. T. Bastos, José Luiz M. do Nascimento, Edilene O. Silva

**Affiliations:** Universidade Federal do Pará, Instituto de Ciências Biológicas, Laboratório de Parasitologia e Laboratório de Biologia Celular, Avenida Augusto Corrêa, 01, Bairro Guamá, 660975-110 Belém, Pará Brazil; Universidade Federal do Pará, Instituto de Ciências Exatas e Naturais, Laboratório de Cromatografia Líquida, Avenida Augusto Corrêa, 01, Bairro Guamá, 660975-110 Belém, Pará Brazil; Universidade Federal do Pará, Instituto de Ciências Biológicas, Laboratório de Neuroquímica Molecular e Celular, Avenida Augusto Corrêa, 01, Bairro Guamá, 660975-110 Belém, Pará Brazil; Instituto Nacional de Ciência e Tecnologia em Biologia Estrutural e Bioimagem, Ilha do Fundão, 21941 902 Rio de Janeiro, Rio de Janeiro Brazil; Instituto Evandro Chagas, Laboratório de Microscopia Eletrônica, SVS, MS, Av. Almirante Barroso 492, Bairro Marco, 66090-000 Belém, Pará Brazil; Universidade Federal do Pará, Laboratório de Biologia Estrutural, Avenida Augusto Corrêa, 01, Bairro Guamá, 660975-110 Belém, Pará Brazil

**Keywords:** *Physalis angulata*, Aqueous extract, Physalins, *Leishmania amazonensis*, Morphological alterations, Macrophage viability, Antileishmanial activity

## Abstract

**Background:**

Leishmaniasis is an infectious disease caused by various species of the protozoan parasites of the *Leishmania* genus and transmitted by phlebotomine sandflies. The protozoa multiply in phagocytic cells, mainly macrophages, which play an important role defending the organism from pathogens. The most effective treatment for leishmaniasis is the chemotherapy and besides the high cost, these drugs are toxic and require a long period of treatment. Currently, some herbal products are considered an important alternative source of a new leishmanicidal agent, which includes the plant *Physalis angulata*, . We evaluated effects of an aqueous extract from roots of *Physalis angulata* (AEPa) on *Leishmania* proliferation, morphology and also determined whether physalins were present in the extract contributing to the knowledge of its pharmacological efficacy.

**Methods:**

Morphological alterations were determined by light microscopy, transmission and scanning electron microscopy. Host cell viability was evaluated by MTT, and propidium iodide. AEPa were submitted in full HRESITOF analysis.

**Results:**

AEPa promoted a dose-dependent reduction on promastigotes (IC_50_ = 39.5 μg/mL ± 5.1) and amastigotes (IC_50_ = 43.4 μg/mL ± 10.1) growth. This growth inhibition was associated with several morphological alterations observed in promastigote forms. No cytotoxic effect in mammalian cells was detected (IC_50_ > 4000 μg/mL). Furthemore, the presence of physalins A, B, D, E, F, G and H were described, for the first time, in the *P. angulata* root.

**Conclusions:**

Results demonstrate that AEPa effectively promotes antileishmanial activity with several important morphological alterations and has no cytotoxic effects on host cells.

**Electronic supplementary material:**

The online version of this article (doi:10.1186/s12906-015-0717-1) contains supplementary material, which is available to authorized users.

## Background

American tegumentar leishmaniasis (ATL) is a parasitic disease prevalent in many parts of the tropical and subtropical world and affects 12 million people in the entire world [1]. It is estimated that 2 million cases occur worldwide, including more than 22.000 cases in Brazil, with 32 % of them in the Amazon region [1, 2]. ATL is a disease with a wide spectrum of clinical variability that depends on both infecting species of *Leishmania* and the immune response of the host [3].

Chemotherapy is the only effective treatment for this disease. However, it is expensive and usually requires a long-term invasive and toxic intervention. During recent decades, studies have demonstrated that a number of plant-derived chemical compounds may act as new therapeutic tools against *Leishmania* [4–6]. One of these agents is *Physalis angulata* (Solanaceae), a plant distributed in tropical and subtropical regions of the world, including Amazon region that has been used traditionally as folk medicine [7, 8]. River people from the Amazonian usually employ the root from *P. angulata* for therapeutic interventions for malaria, asthma, hepatitis, dermatitis, rheumatism treatment and as anti-inflammatory agent [9–13]. The phytochemistry of *P.angulata* is known to contain glucocorticoids, flavonoids, withanolides and physalins [13]. Among the compounds thought to be responsible for the leishmanicidal effects of *P. angulata* are the physalins, which have been mostly isolated from the plant’s stems and leaves [14, 15]. In addition, antiprotozoal effect of physalins was also been reported against *Plasmodium sp*. [16] and *Trypanosoma cruzi* [17]. Nevertheless, to isolate enough physalins to observe the above-mentioned effects, researchers have used large quantities of *P. angulata* leaves and/or stems. As the purification process is time, effort, and resource consuming, the trend nowadays is to determine whether or not effective therapeutic results can be achieved from plant extracts, once extracts and/or infusions from *P. angulata* are most commonly used in popular medicine as a treatment for those infirmities [7, 8, 12, 13]. To date, no study has assessed whether extracts from *P. angulata*’s root affect *Leishmania* parasites, the main host cell (macrophage), or determined the presence of physalins in the root extract.

Therefore, based in effective action of *P. angulata* against *Leishmania* parasites, we reported here, for the first time, ultrastructural alterations in promastigote forms of *Leishmania (Leishmania) amazonensis* and host cell viability after exposure to aqueous root extract obtained from *P. angulata*. In addition, we also determined the presence of physalins in the root extract.

## Methods

### Plant materials

*P. angulata* of the family Solanaceae was collected in the Pará state, Brazil, and identified by Dr. Ricardo Secco (Department of Botany, Emilio Goeldi Museum) and a voucher specimen (no. 653) was deposited in the herbarium of the Emilio Goeldi Museum (Belém, Pará, Brazil). Extraction process were carried out according to the method described by Bastos et al. [11] and 1.0 mg/mL of aqueous extract from root of *Physalis angulata* (AEPa) was dissolved in Dulbecco’s Modified Eagle’s Medium (DMEM) or RPMI and used as the standard solution for assays.

### Liquid chromatography/time-of-flight mass spectrometry analysis

High resolution mass spectral (HRESITOF) analysis was performed on a micrTOF II - ESI-TOF (Brucker Daltonics, Billerica, MA, USA). The capillary voltage was monitored at 4500 V (120 V output). A solution of NA-TFA (10 mg/mL) was used for Internal calibration (TOF). N_2_ was used at 200 °C; flow and pressure were set at 4 L/min nebulization and 0.4 bar. Liquid chromatography was performed with a Prominence liquid chromatography system model LC-20A from Shimadzu Technology (Kyoto, Japan), fitted with a diode array detector (DAD), SPDM-20A (Shimadzu, Kyoto, Japan) working in the range of 220–250 nm, a two pump LC-20 AD (Shimadzu, Kyoto, Japan), and a DGU-20A-5 degasser; a CBM-20A interface was used (Shimadzu, Kyoto, Japan). The analysis was performed at room temperature. The optimal chromatographic conditions consisted of an isocratic solvent system containing 25 % of acetronitrile in water, delivered to the C18 Phenomenex Onyx Monolithic 3 μm column (100 mm × 4.6 mm I.D.) at a flow rate of 0.3 mL/min. The eluent was monitored at a 225 nm detection wavelength.

### Parasites

*Leishmania (Leishmania) amazonensis* (IFLA/67/BR/PH8) promastigotes were obtained from the Evandro Chagas Institute and cultured at 26 °C in NNN medium. Subsequently, promastigotes were cultured in RPMI medium supplemented with 10 % fetal bovine serum (FBS), 0.2 M glutamine, 0.125 M pyruvic acid and 5 mM penicillin/streptomycin.

### Isolation of peritoneal murine macrophages

Females BALB/c mice (6 to 8 weeks old) were euthanized and resident macrophages were obtained by washing the peritoneal cavities with DMEM, pH 7.2. Then, peritoneal macrophages were allowed to adhere on round glass coverslips placed 24-well culture plate in a DMEM medium supplemented with 10 % FBS for 1 h at 37 °C in a humidified atmosphere containing 5 % CO_2._ After that, non-adherent cells were washed away with phosphate buffered saline (PBS), pH 7.2, and macrophages were incubated overnight in DMEM medium supplemented with 10 % FBS at 37 °C in a 5 % CO_2_ atmosphere. All experiments were performed at least three times with treated and untreated cells. The experiments were conducted in compliance with the Brazilian animal protection law (Lei Arouca number 11.794/08) of the National Council for the Control of Animal Experimentation (CONCEA, Brazil). The protocol was approved by the Committee on the Ethics of Animal Experiments of the Federal University of Pará (CEPAE/ICB/UFPA - grant number BIO086-12).

### Antipromastigote assay

*L. (L.) amazonensis* promastigotes (10^6^ parasites/mL) in the logarithmic growth phase were inoculated in a 24-well plate containing RPMI medium supplemented with 10 % FBS with different concentrations of AEPa, and incubated at 25 °C. Every 24 h, aliquots were harvested and promastigotes were counted in a Neubauer chamber. Cells were counted daily up to 96 h of treatment. The cultures were performed in triplicate. One known antileishmanial drug (Amphotericin B) at 0.1, 0.25, 0.5 and 1 μg/mL was included as a positive control. The inhibitory concentration (IC_50_) was determined using SigmaPlot (version 12).

### Antiamastigote assay

Adhered macrophages were infected with *L. (L.) amazonensis* promastigotes (stationary growth phase) at a parasite/macrophage ratio of 10:1 and incubated for 3 h at 37 °C and 5 % CO_2_. After 1 h, free parasites were removed by washing with PBS. Cells were treated with 20, 50 and 100 μg/mL of AEPa, respectively, for 1 h daily and incubated for 3 days post infection, replacing the culture medium. Cells were then washed with PBS, fixed with Bouin’s fixative, stained with Giemsa and covered with Entellan® (Merck®). The number of parasites was determined by examining three coverslips for each treatment. At least 200 infected macrophages were counted and results were expressed as survival index when compared to controls. The survival index was obtained by multiplying the percentage of the infected macrophages by the mean number of amastigotes per infected macrophage and divided by the total number of macrophages. The percentage of inhibition was determined based in the survival index compared with control. The inhibitory concentration (IC_50_) was determined using SigmaPlot (version 12). One known antileishmanial drug Glucantime (50 μg/mL) was included as a positive control.

### Morphological Analysis of AEPA-treated *L. (L.) amazonensis* promastigotes

#### Light microscopy (LM)

To evaluate the morphological changes induced by the AEPa in promastigote forms of *L. (L.) amazonensis*, the parasites were treated with 50 and 100 μg/mL of AEPa for 72 h. Parasite suspension aliquots were fixed with 4 % paraformaldehyde in 0.1 M PHEM buffer (5 mM magnesium chloride, 70 mM potassium chloride, 10 mM EGTA, 20 mM HEPES, 60 mM PIPES), pH 7.2, and stained with Panotic stain Kit (Laborclin). Cells were examined using an optical microscope Axioplan (Zeiss) and images were obtained using a digital camera model XC 30.

#### Transmission Electron Microscopy (TEM)

*L. (L.) amazonensis* promastigotes (10^6^) in the logarithmic growth phase (4 days) were treated with 50 and 100 μg/mL AEPa for 3 days. The samples were processed for conventional TEM, as described by Guimarães et al. [18].

#### Scanning Electron Microscopy (SEM)

Control and treated promastigotes (50 and 100 μg/mL AEPa) were fixed with 4 % formaldehyde and 2.5 % glutaraldehyde in 0.1 M cacodylate buffer, pH 7.2, for 1 h. After fixation, the cells were placed on glass slides with 0.1 % poly-L-lysine. The cells were washed and post-fixed in 1 % osmium tetroxide, dehydrated in graded ethanol, critical point dried (CO_2_), coated with platinum and examined with a LEO 1450VP SEM.

### Murine macrophage viability assays

#### MTT assay

The MTT assay is based on the mitochondrial-dependent reduction of MTT 3-(4,5-dimethylthiazol-2-yl)-2,5-diphenyl tetrazolium bromide] to formazan, and this assay was conducted following the procedure described by Rodrigues et al. [19]. Macrophages were treated with AEPa (10–400 μg/mL) for one hour and cultured at 37 °C, 5 % CO_2_ for 24 h. After treatment, cells were incubated with 0.5 mg/mL MTT for 3 h. Subsequently resulting solution was read in a microplate reader (BIO-RAD Model 450 Microplate Reader) and absorbance was recorded at 570 nm.

#### Propidium iodide dye exclusion test

Control and treated macrophages (50 and 100 μg/mL AEPa) were incubated for 30 min with 10 μg/mL of propidium iodide (PI). A total of 10.000 events were acquired for each sample in the region that corresponded to the parasites by flow cytometer BD FACSCantoII and analyzed with BD FACS Diva software. Cells labeled with the PI were considered dead.

### Statistical analysis

The differences between mean values in the experimental groups were performed using ANOVA followed Tukey’s post test. All P-values < 0.05 were considered statistically significant. The means and SD of at least three experiments were determined.

## Results

### Identification of AEPa components by mass spectrometry analysis

To identify the possible active principle(s) responsible for the biological and morphological effects described above, we submitted AEPa to HRESITOF analysis. Several peaks were observed in the eluent from the liquid chromatography column. Among them, we identified the physalins A, B, D, E, F, G and H (Table 1).

**Table 1:** High resolution mass spectral analysis of AEPa compounds performed on a micrTOF II - ESI-TOF

Compound	Substance	T_r_	Theoretical *m*/*z*	Experimental *m/*z
1	Physalin D	2.35	545.2023	545.2021
2	Physalin G	2.55	527.1917	527.1902
3	Physalin A	3.22	527.1917	527.1902
4	Physalin F	7.03	527.1917	527.1902
5	Physalin E	7.94	545.2023	545.2021
6	Physalin H	8.99	563.1684	563.1684
7	Physalin B	13.17	511.1968	511.1968

*tR* time retention

### Antileishmanial activity of AEPa

**Fig. 1:**
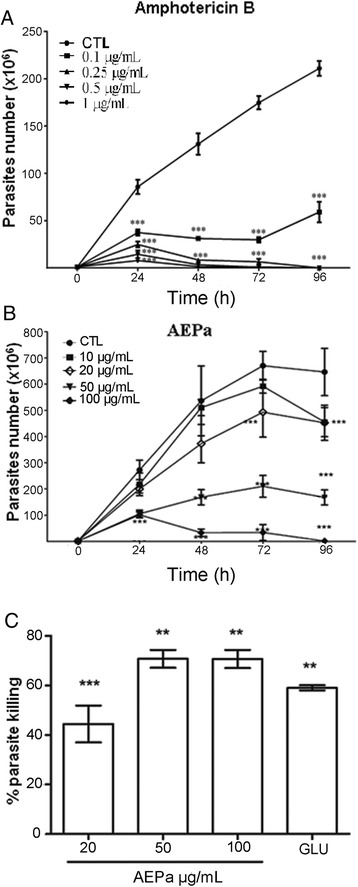
AEPa action against *L. (L.) amazonenis in vitro*. Growth curve of *L. (L.) amazonensis* promastigotes treated with different concentrations of Amphotericin B (AMPB) (**a**) and AEPa. **b** Growth was followed up to 96 h. **c** Effect of AEPa on intracellular amastigote survival of *L. (L.) amazonensis.* Infected mouse peritoneal macrophages were treated with different concentrations of the extract, for1h/day during 3 days and with glucantime ® (GLU). Results are from experiments performed in triplicate. *p < 0.001, *p < 0.05, ***p < 0.001 compared with control (untreated). CTL: control

The effect of AEPa on *L. (L.) amazonensis* promastigotes was monitored for 4 days. Reductions of 100 % were observed in the number of viable parasites at 0.5 and 1 μg/mL Amphotericin B, respectively, after 96 h of incubation, representing an IC_50_ of 0.02 μg/mL ± 0.021 (Fig. 1a). In contrast, the AEPa induced dose-dependent reductions in parasite proliferation of 74.1 % and 99.8 % after 96 h of treatment with 50 μg/mL and 100 μg/mL AEPa, respectively, representing an IC_50_ of 39.5 μg/mL ± 5.1 μg/mL (Fig. 1b).

### Effect of AEPa on *L. (L.) amazonensis* intracellular amastigotes

The leishmanicidal activity of AEPa was also evaluated in *L. (L.) amazonensis*-infected macrophage cultures. A reduction in parasite survival was observed for all AEPa concentrations tested. However, more significant results were found at 50 μg/mL and 100 μg/mL of AEPa, achieving reductions of 70.6 % and 70.9 %, respectively (IC_50_ 43.3 μg/mL ± 10.1; Fig. 1c). Glucantime (GLU - Figure 1c) and amphotericin B (AMPB - Additional file 1) were added as reference drugs.

### LM analysis of AEPa-treated *L. (L.) amazonensis* promastigotes

**Fig. 2:**
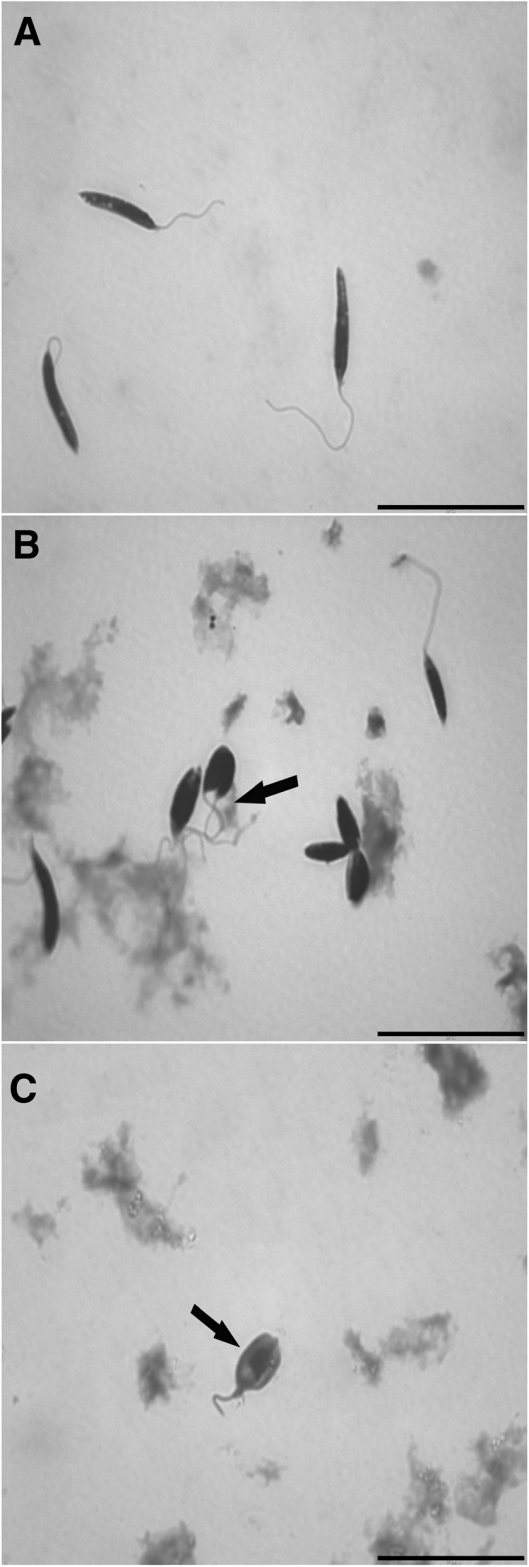
Light microscopy of promastigote forms of *L. amazonensis* treated with AEPa for 3 days. **a** Control parasite; **b** Promastigotes treated with 50 μg/mL of AEPa. Arrow indicates parasites with two flagella. **c** Promastigotes treated with 100 μg/mL AEPa. Note reduction in the cell size and rounded cell body (arrow). Bars: 20 μm

*L. (L.) amazonensis* promastigotes were analyzed by LM to detect morphological alterations. Untreated parasites showed a typical morphology with an elongated body and a single flagellum (Fig. 2a). After 72 h of treatment with 50 μg/mL and 100 μg/mL of AEPa, promastigotes presented a rounded shape, two flagella and a reduction in cell body (Fig. 2b and c). In addition, treated cultures presented debris suggestive of cellular fragments.

### SEM analysis of AEPa-treated *L. (L.) amazonensis* promastigotes

**Fig. 3:**
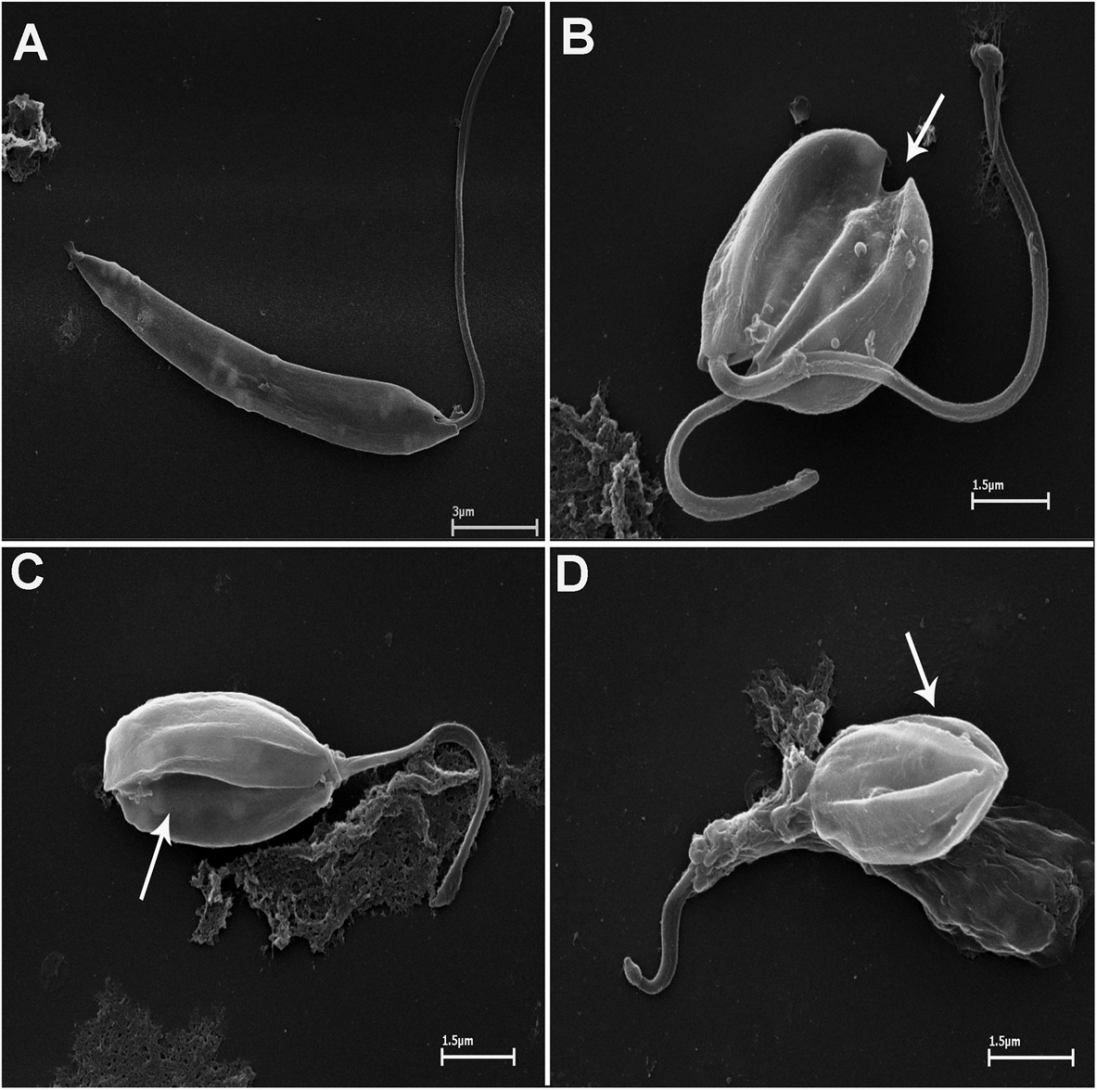
Scanning electron microscopy of promastigotes of *L. amazonensis* treated with AEPa from *Physalis angulata* after incubation for 3 days. **a** Control. **b** Parasites after treatment with 50 μg/mL AEPa. Observe the flagellum and cell doubling in apparent atypical cell division, and multi-septation of the cell body (*arrows*). **c** and **d** Parasites after treatment with 100 μg/mL AEPa. Observe reduction in the parasite body, multi-septation of the cell body (white arrows) and of the flagellum. Note the presence of cellular debris around parasite (*arrows*). Bars: (**a**) 3 μm (**b**-**d**) 1.5 μm

SEM was used to further investigate the morphological alterations observed by light microscopy. Promastigotes treated with 50 μg/mL AEPa presented flagellum duplication and atypical cell division (Fig. 3b). When promastigotes were treated with 100 μg/mL AEPa, their rounded appearance became more evident, the body was frequently multi-septated, and the flagellum shortened (Fig. 3c and c). Moreover, SEM analysis suggest that the debris observed by LM is likely to be cellular fragments around the parasites (Fig. 3d).

### TEM analysis of AEPa-treated *L. (L.) amazonensis* promastigotes

**Fig. 4:**
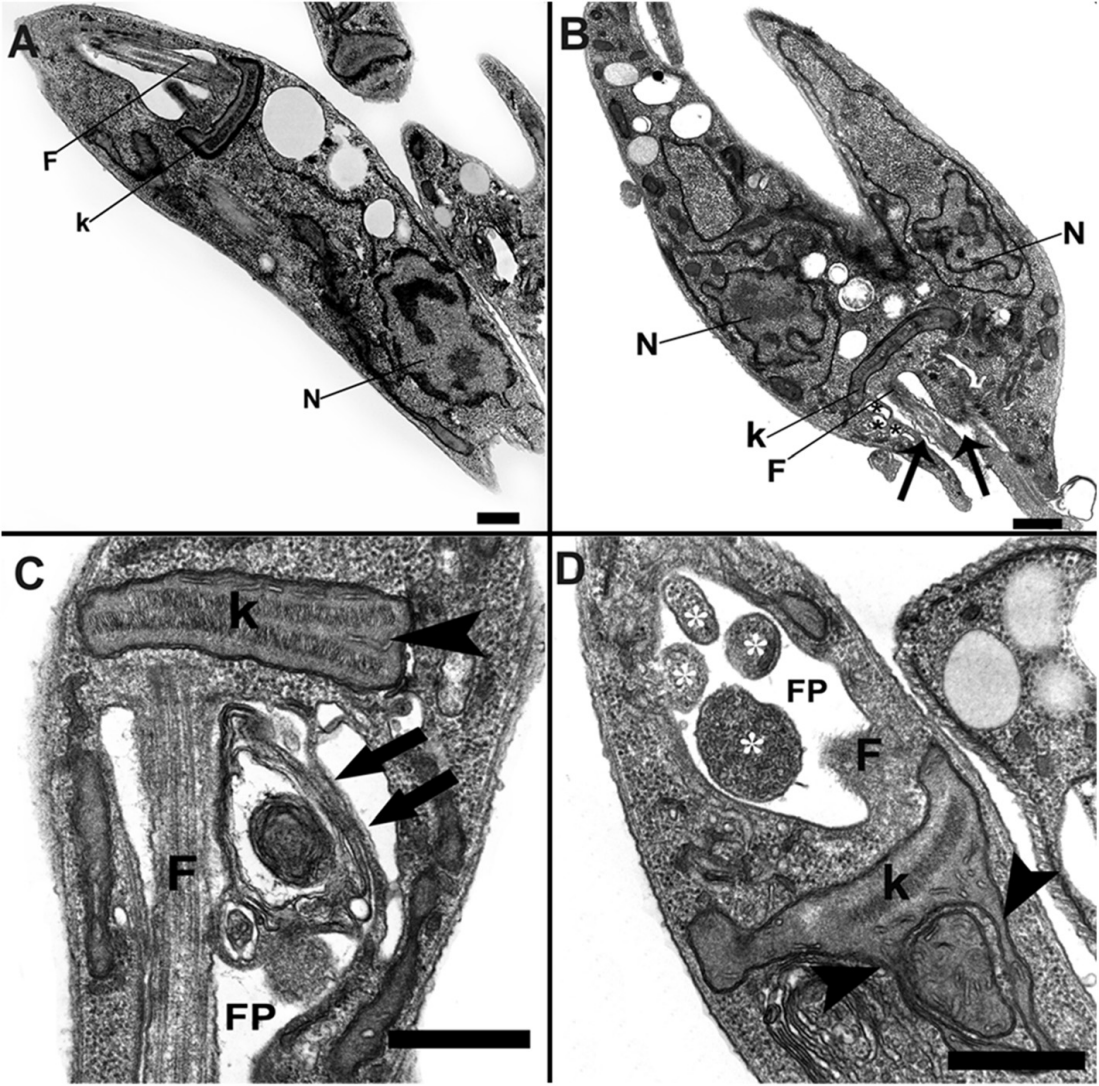
Ultrastructural effects of AEPa on promastigotes of *Leishmania (L.) amazonensis*. **a** General view of untreated parasite showing the characteristic structure of kinetoplastids. **b** Promastigotes treated with 50 μg/mL AEPa for 3 days. Note some vacuoles in the membrane of flagellar pocket (*) and alterations in flagellar membrane (*arrow*). **c** and **d** General view of promastigotes treated with 100 μg/ml AEPa for 3 days. **c** Observe the presence of myelin-like structures in the flagellar pocket (*arrows*) and duplication of kinetoplast DNA (arrowhead). (**d**) Note the presence of a large number of vesicles inside the flagellar pocket (*) and alterations in shape and swelling of kinetoplast (*arrowheads*). *N*, nucleus; *FP*, flagellar pocket; *K*, kinetoplast; *F*, flagellum; *M*, mitochondria. Bars represent A 5 μm; B 2 μm; C 5 μm; C *inset* 2 μm; D 2 μm

TEM was used to analyze alterations in the parasite organelles. Promastigotes treated with 50 μg/mL AEPa showed changes in the flagellar membrane that presents some vacuoles near the flagellar pocket membrane (Fig. 4b). When 100 μg/mL AEPa was used, we observed alterations in the kinetoplast’s shape with swelling and duplication of its DNA. Alterations were also observed in the flagellar pocket, where myelin-like structures and the presence of vesicles were seen (Fig. 4c and d). Furthermore, atypical cell division was observed (Fig. 4b), as shown by SEM.

### Effects of AEPa on macrophage viability

**Fig. 5:**
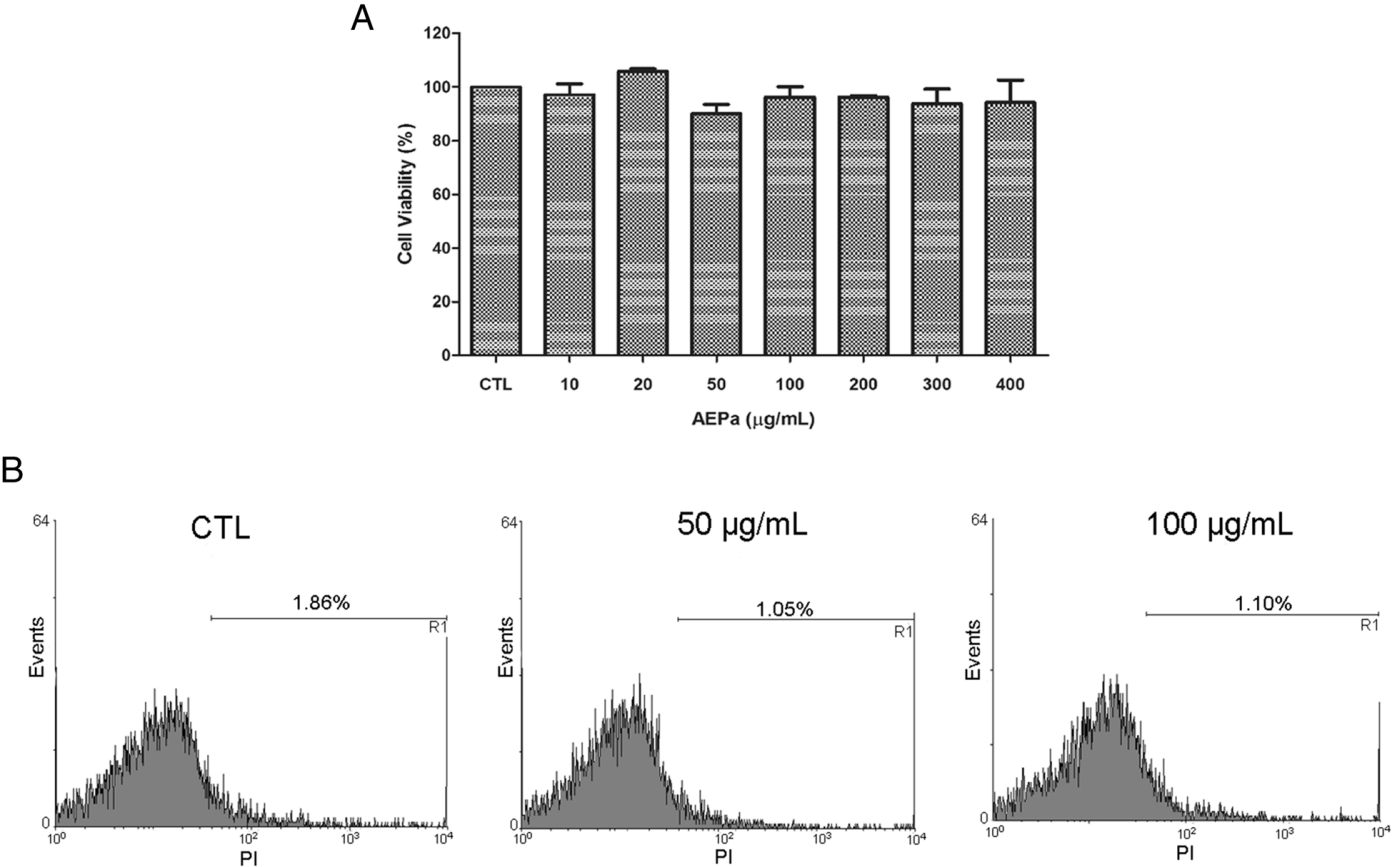
Host cell viability assays. **a** MTT assay. The viability of the untreated control was taken as 100 %. The viability percentage was calculated for different concentrations of AEPa. No significant differences were found at 10–400 μg/ml of AEPa when compared with the control. **b** Propidium iodide (PI) exclusion test at 50 and 100 ug/mL in comparison to the control (CTL). No significant differences were observed in macrophages stained with PI dye

To test the selectivity of AEPa for the *L. (L.) amazonensis* parasite, murine macrophages were treated with the extract and the viability of these cells was assessed by the MTT reduction and PI. No cytotoxic effect of 10–400 μg/mL of AEPa treatment on macrophages was observed with the MTT assay (Fig. 5a). No differences were detected in the intensity of the red fluorescence in macrophages stained with PI dye (Fig. 5b) when compared treated and untreated macrophages.

## Discussion and conclusions

Bioproducts obtained from plants offer perspectives for the discovery of new active and promising compounds with antileishmanial activity. Extracts and physalins obtained from *P. angulata* have shown a vast range of biological properties [10, 11, 14–16, 20, 21]. However, to date, no reports have described the morphological alterations in parasites of *Leishmania* genus in response to AEPa treatment.

Results showed that 100 μg/mL AEPa induce 99.8 % antileishmanial activity against *L. amazonensis* promastigotes (IC_50_ of 39.5 μg/mL ± 5.1). A similar concentration of flower and leaf extracts from *Cymbopogon citratus, Matricaria chamomilla* and *Piper regnellii* Miq. [5] achieved around 98 % antileishmanial activity against *L. amazonensis* promastigotes. As such, AEPa seems to have a comparable growth inhibitory effect on promastigotes to these other plant extracts. However, they are clearly more potent than some plant extracts that have reported a maximum of 90–96 % of antileishmanial activity against *L. amazonensis* promastigotes [5, 22].

One of the novel findings of the present study is the description of morphological alterations caused in promastigotes forms following incubation with AEPa. Electron microscopy techniques can be a useful tool in drug studies for the identification of cell surfaces and ultrastructural alterations, as well as specific organelle targets in the parasites. Indeed, identification of morphological changes can help elucidate the mechanisms of drug action [23, 24]. Treatment of promastigotes with 50 or 100 μg/mL AEPa caused a reduction and rounding of the body cellular volume, as well as flagellum duplication, as shown by LM and SEM. Duran et al. [25] observed similar changes in the body shape and flagellum of promastigotes treated with an ethanolic extract of *Adana propolis*. However, these authors used concentrations of 250 and 500 μg/mL against *L. tropica*. Santin et al. [26] showed atypical flagella duplication in *L. amazonensis* promastigotes treated with oil from *Cymbopogon citratus*. In a review published by Adade and Souto-Padrón. [24], the authors suggested that these alterations may be caused by the interference of drugs and plant extracts in the process of cell division. Since the division process of *L. amazonensis* promastigotes treated with AEPa initiated at the posterior instead of the normal anterior position [27, 28], it is possible that the extract interfered with the division process of these cells.

SEM analysis revealed cellular debris and body multi-septation in promastigotes treated with AEPa. Studies with *Cymbopogon citratus* oil against promastigotes of *L. chagasi* showed multi-septation, and shortening of the cell body and flagellum [29]. This alteration could be caused by cytoskeleton disassembly [24]. In addition, TEM analysis showed the presence of multiple vesicles within the flagellar pocket of AEPa treated-promastigotes. The uptake of macromolecules by promastigotes occurs through the flagellar pocket, which is an invagination of the plasma membrane, devoid of microtubules. This region is the only place where exocytic and endocytic activity occurs [30]. In promastigotes submitted to drug and/or plant extracts treatments, this increased exocytic activity probably occurs to expel the toxic compound [18, 31]. It is likely that the multiple vesicles observed may be employed by *L. amazonensis* promastigotes for the removal of the toxic compounds present in the AEPa.

Myelin-like figures were also observed in the promastigotes treated with AEPa. These structures are a result of the merging of internal membranes to form concentric structures, which may be indicative of an autophagic process [18, 32, 33] as observed by Meira et al. [17] in *Trypanosoma cruzi*. Another characteristic feature seen in AEPa-treated promastigotes was the structural modifications of the kinetoplast, such as duplication, and shape and size alterations. Several authors, using plant products, demonstrated similar kinetoplast alterations [18, 34]. The kinetoplast is a unique organelle present only in trypanosomatid protozoa and is, thus, a potential drug target due to its unique structure and function, which is matched in its mammalian host [24, 30, 35].

Additionally, treatment of intracellular amastigotes of *Leishmania (L.) amazonensis* with 100 μg/mL AEPa led to a growth inhibitory effect of 70.9 % (IC_50_ 43.3 μg/mL ± 10.1). Luize et al. [5] also showed an inhibitory effect of 92–96 % with *Cymbopogon citratus, Matricaria chamomilla* and *Piper regnellii* Miq. extracts when tested against amastigotes. However, it is not possible to directly compare the inhibitory effects of this study with our results, since authors used axenic amastigote cultures instead of amastigotes inside host cells. In previous reports, Guimarães et al. [14, 15] showed that physalins B, F and G, purified from stems of *P. angulata,* were able to reduce promastigote (IC_50_ = 6.8, 1.4, and 9.2 μM, respectively) and axenic amastigotes number (IC_50_ = 0.21 and 0.18 μM for Physalin B and F, respectively) and percentage of *Leishmania*-infected macrophages at concentrations non-cytotoxic to macrophages. However, no reports have described morphological alterations in parasites. Our study evaluated the antileishmanial effect of AEPa and also morphological alterations indicative of abnormalities in the cell division process. We detected, for the first time, the presence of physalins A, B, D, E, F, G and H in the AEPa, isolated from roots, and believes that these morphological and physiological changes could be related to the presence of these physalins in the AEPa. Furthermore, cytotoxicity tests were employed to verify whether AEPa was selectively toxic against the parasite, therefore sparing the host cell. The assays used did not detect significant changes in host cell viability, suggesting AEPa was specific for the protozoa.

Taken together, our results indicate that the AEPa promotes morphological alteration in promastigotes*,* a dose-dependent reduction of promastigotes and amastigotes of *Leishmania,* and have no cytotoxic effects on the host cell. To our knowledge this report is the first ultrastructural analysis of the actions of the aqueous extract from *P. angulata* on *Leishmania* and could be helpful for understanding possible mechanisms of action of this agent on parasites and host cells. Therefore, this study is part of a continual search for new drugs, obtained from medicinal plants widely used in Amazon region that can act effectively against neglected diseases such as leishmaniasis.

## Additional file

Additional file 1
**Effect of AMPB on** ***L. (L.) amazonensis*** **intracellular amastigotes.** Infected mouse peritoneal macrophages were treated with different concentrations AMPB for 24h. ***p < 0.001 compared with control (CTL).

## Abbreviations

ATL: American tegumentar leishmaniasis
DMEM: Dulbecco’s modified eagle’s medium
HRESITOF: High resolution mass spectral
DAD: Diode array detector
FBS: Fetal bovine serum
AEPa: Aqueous extract from root of *Physalis angulata*
LM: Light microscopy
TEM: Transmission electron microscopy
SEM: Scanning electron microscopy
MTT: 3-(4,5-dimethylthiazol-2-yl)-2,5-diphenyl tetrazolium bromide
PI: Propidium iodide
